# Supplementary figures and images for: A hemoperfusion column selectively adsorbs LAP+ lymphocytes to improve anti-tumor immunity and survival of tumor-bearing rats
Source: PLoS One. 2025 Mar 7;20(3):e0305153. doi: 10.1371/journal.pone.0305153 (PMC11888139; doi:10.1371/journal.pone.0305153)

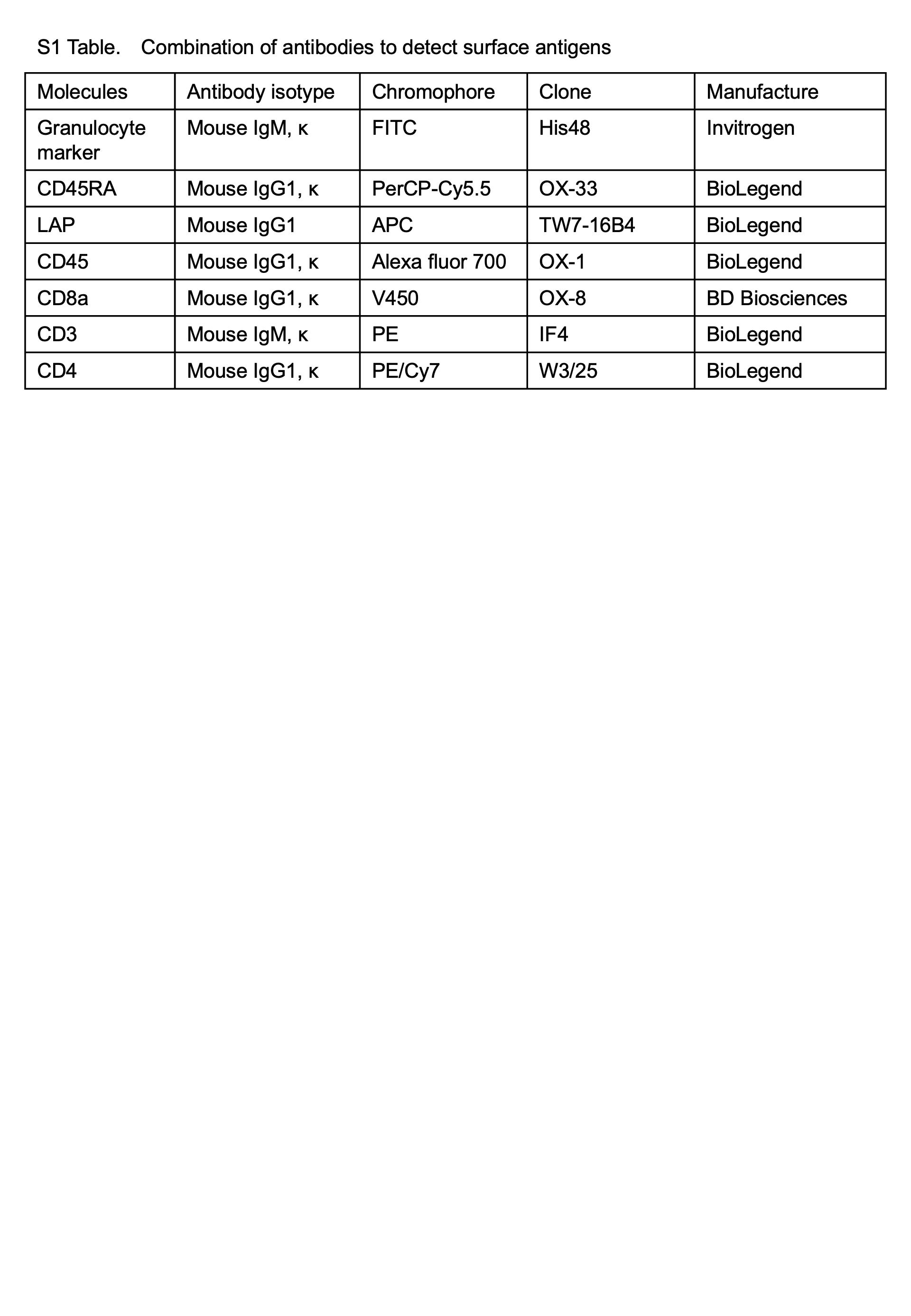

Supplement: S1 Table — (TIFF) [file pone.0305153.s001.tiff]

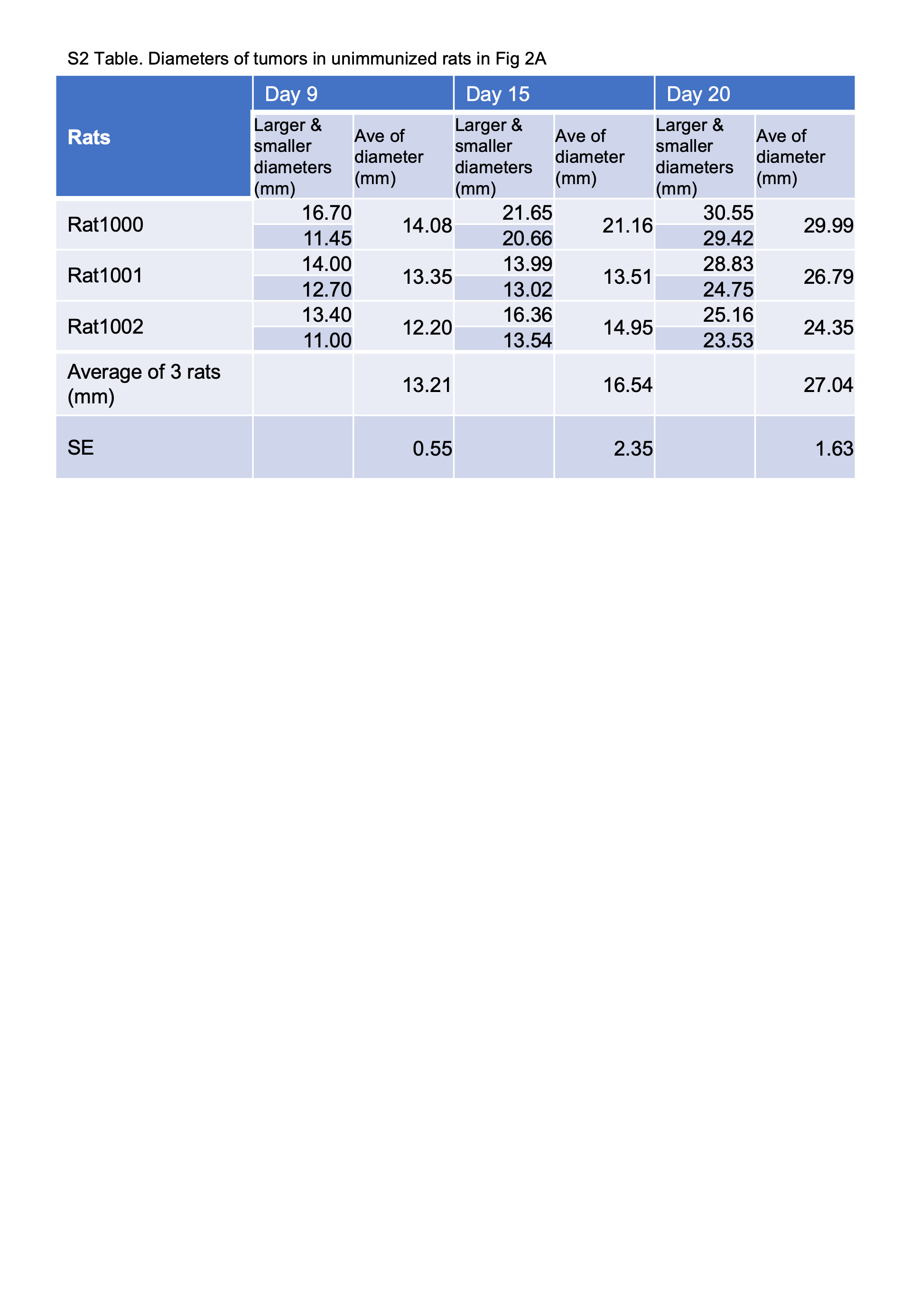

Supplement: S2 Table — (TIFF) [file pone.0305153.s002.tiff]

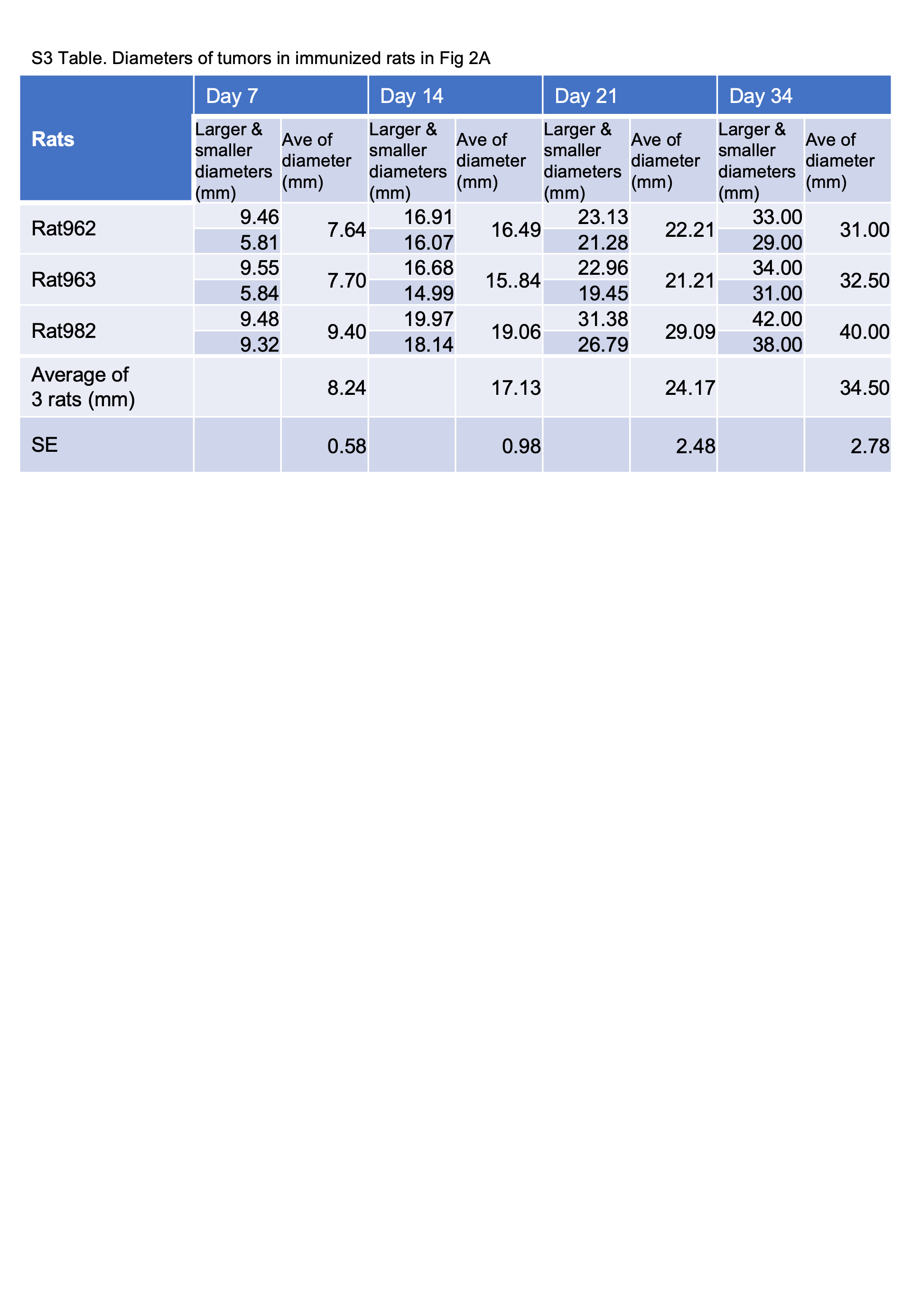

Supplement: S3 Table — (TIFF) [file pone.0305153.s003.tiff]

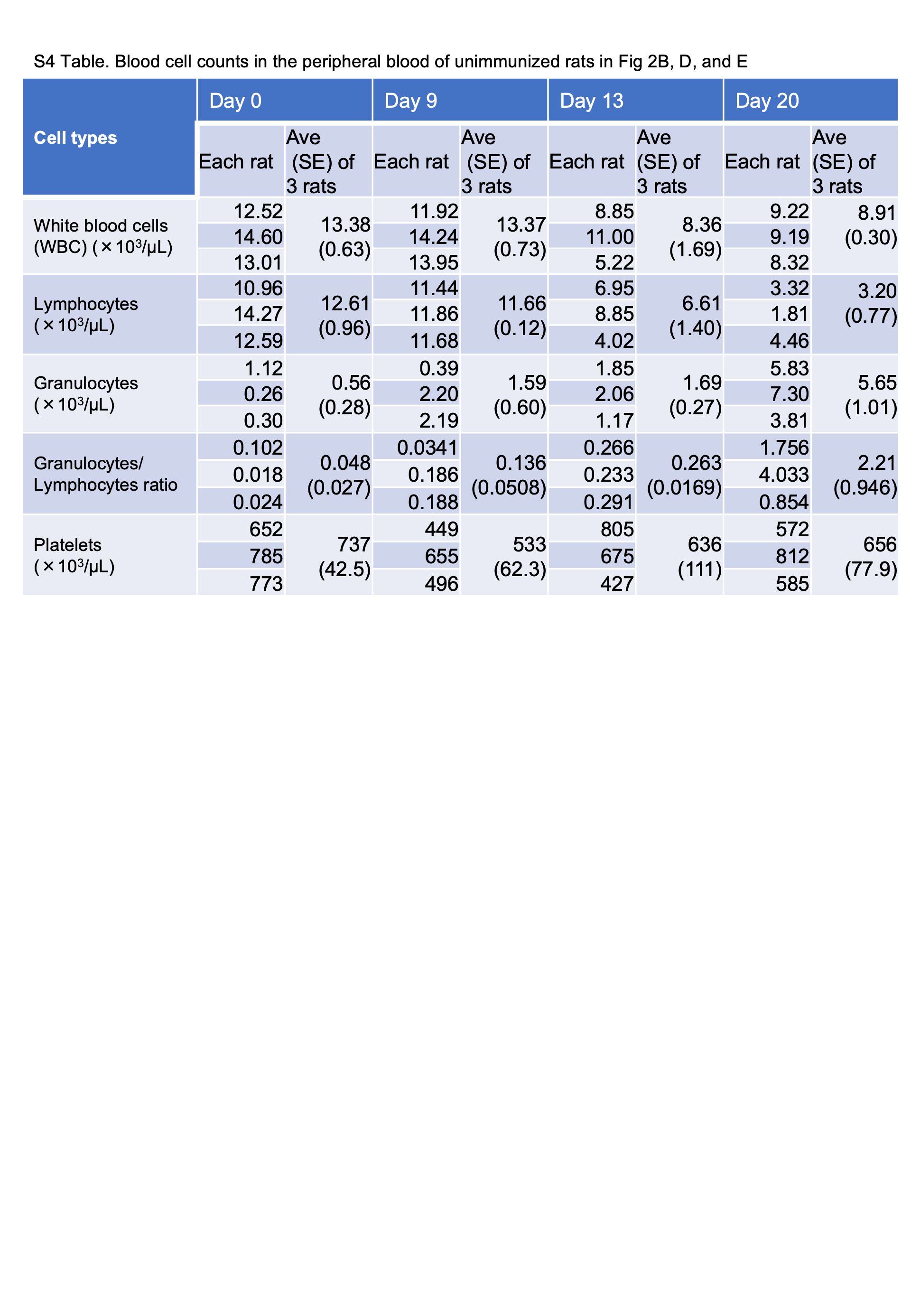

Supplement: S4 Table — (TIFF) [file pone.0305153.s004.tiff]

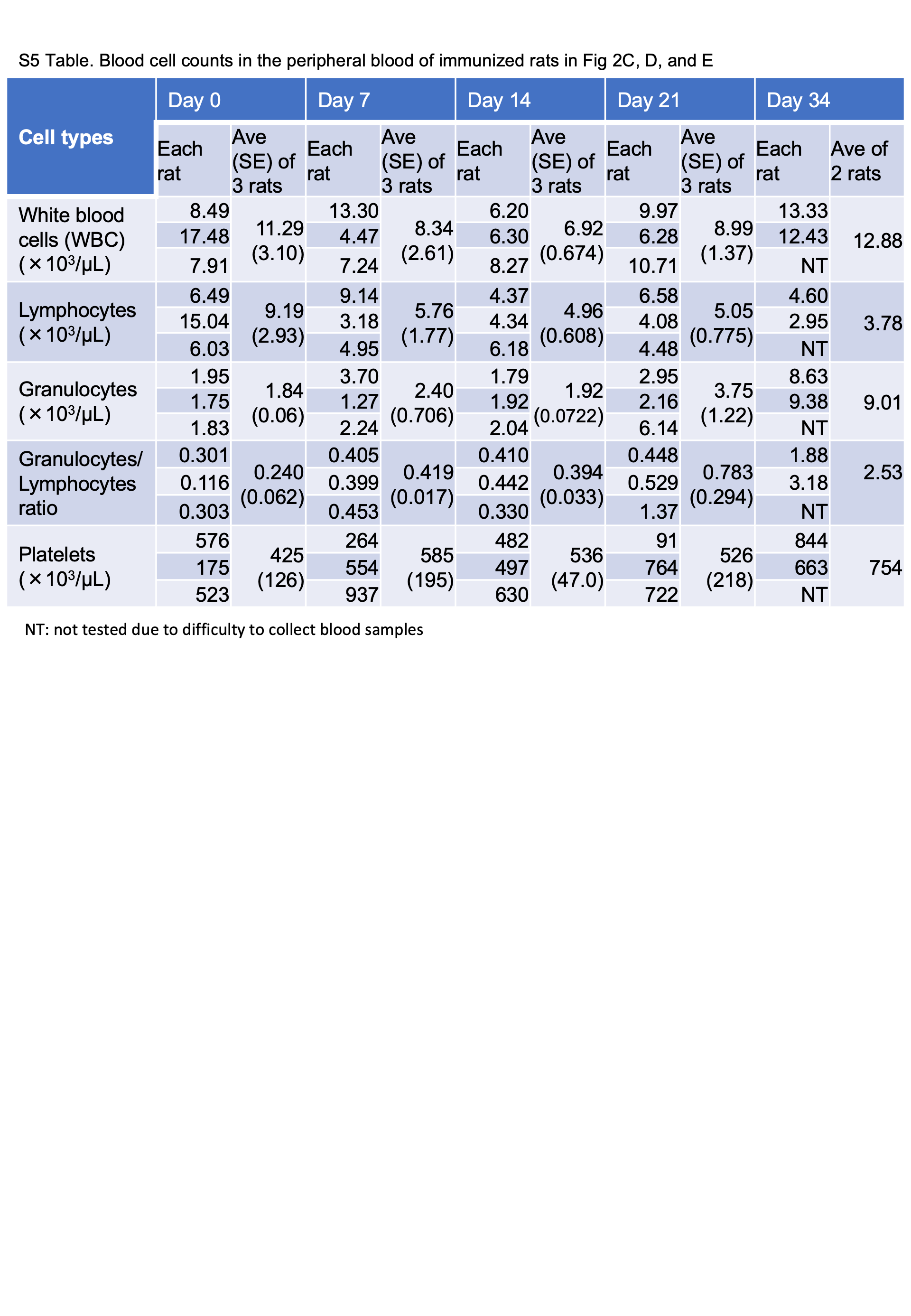

Supplement: S5 Table — NT: not tested due to difficulty to collect blood samples. (TIFF) [file pone.0305153.s005.tiff]

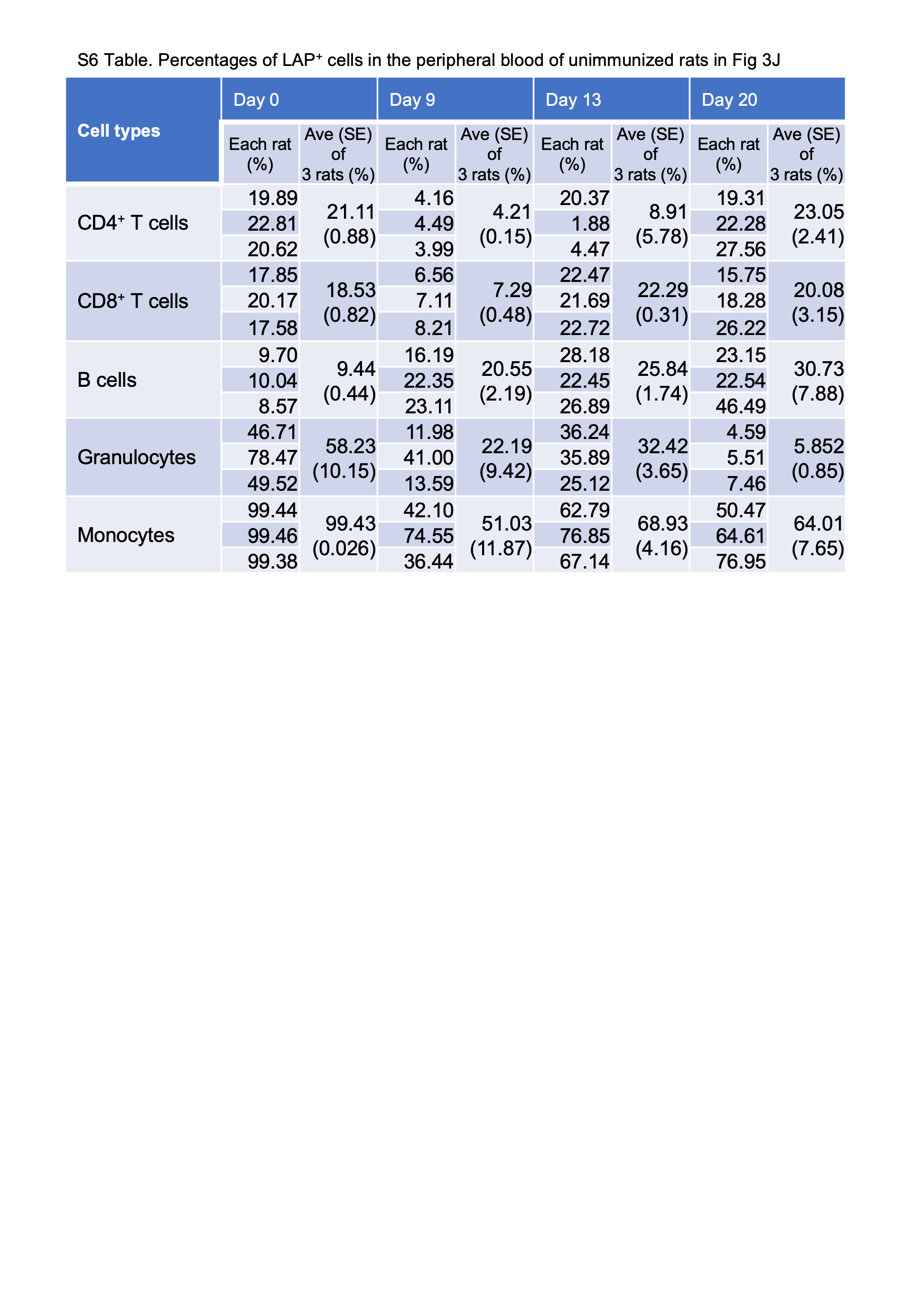

Supplement: S6 Table — (TIFF) [file pone.0305153.s006.tiff]

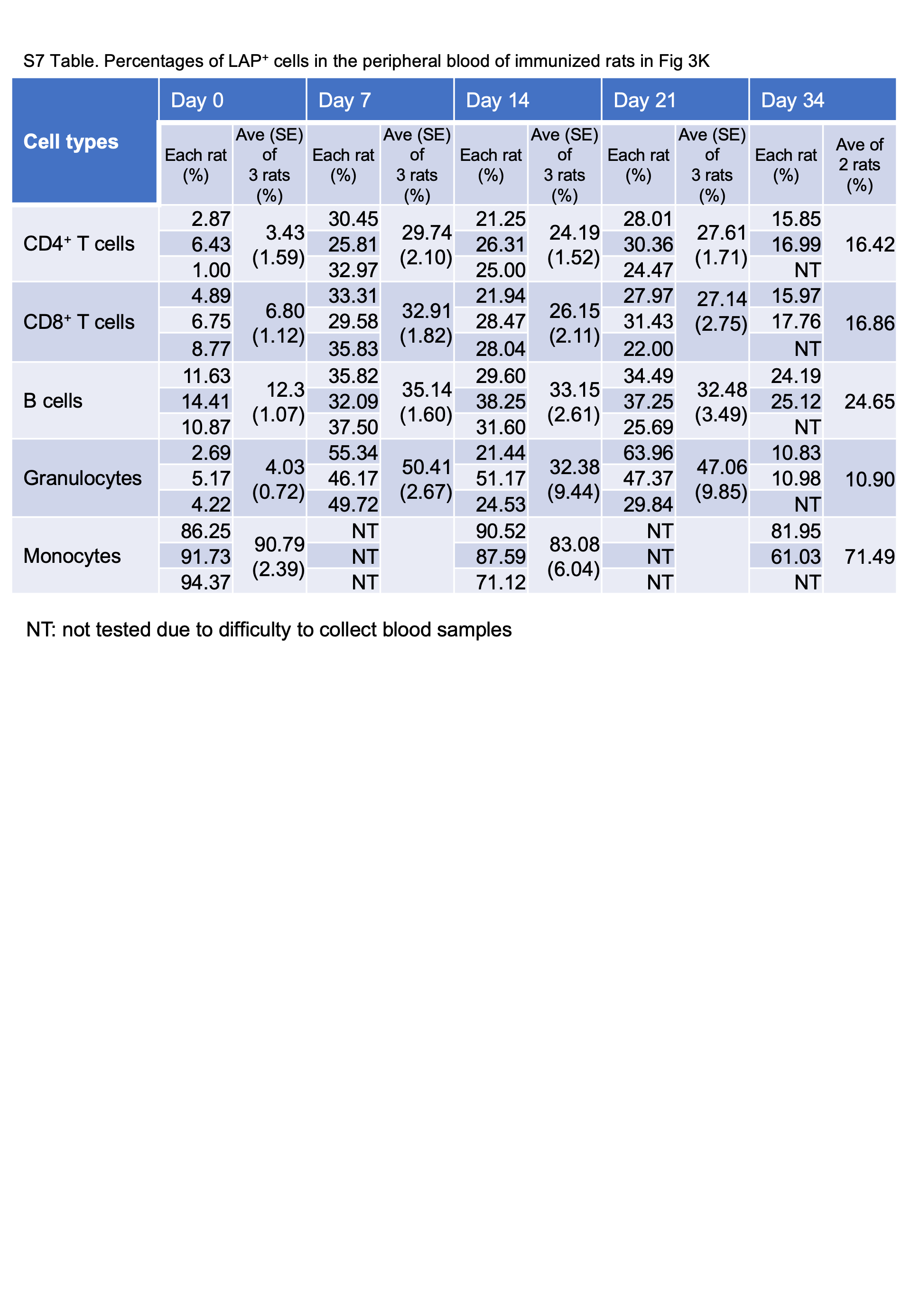

Supplement: S7 Table — NT: not tested due to difficulty to collect blood samples. (TIFF) [file pone.0305153.s007.tiff]

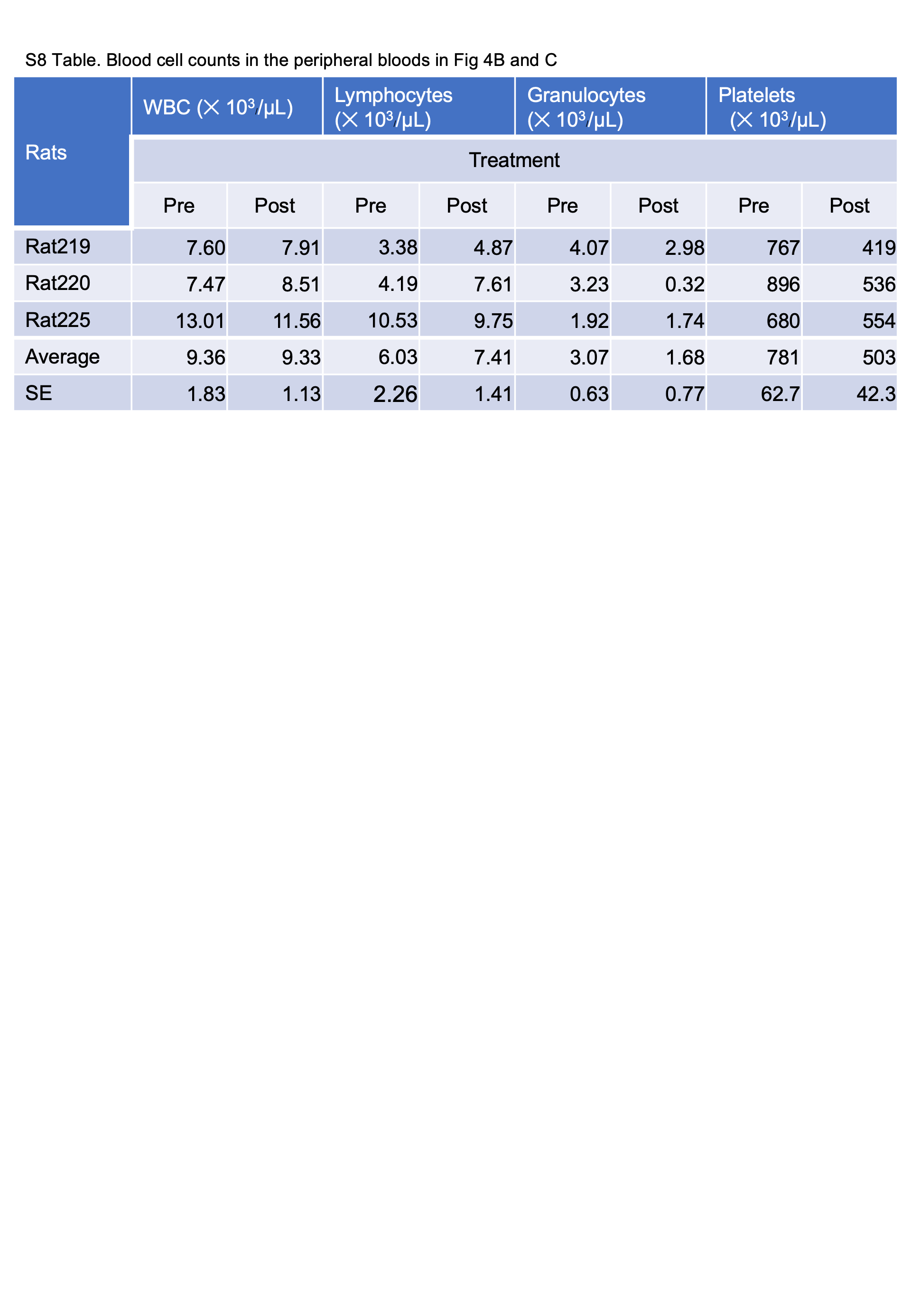

Supplement: S8 Table — (TIFF) [file pone.0305153.s008.tiff]

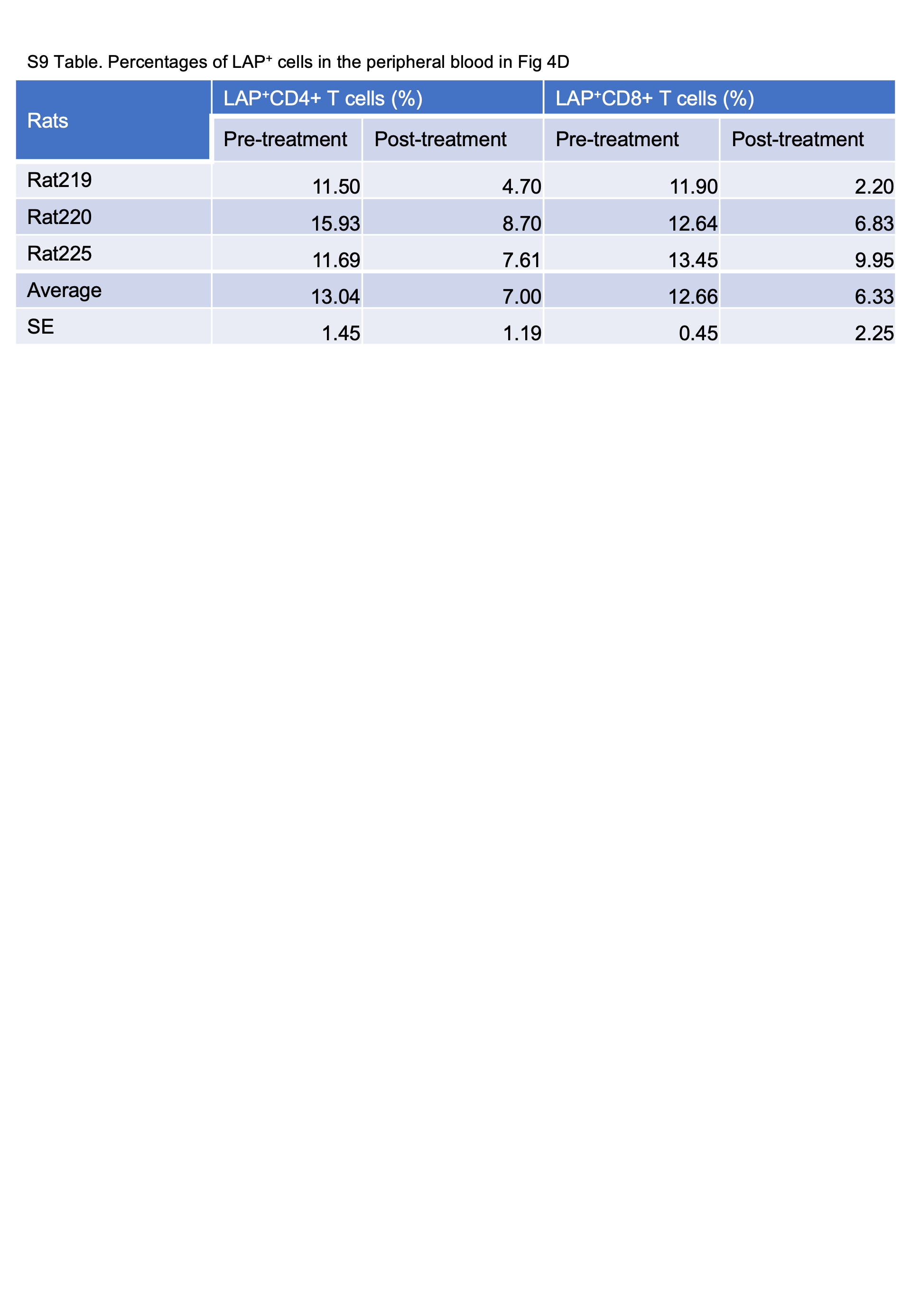

Supplement: S9 Table — (TIFF) [file pone.0305153.s009.tiff]

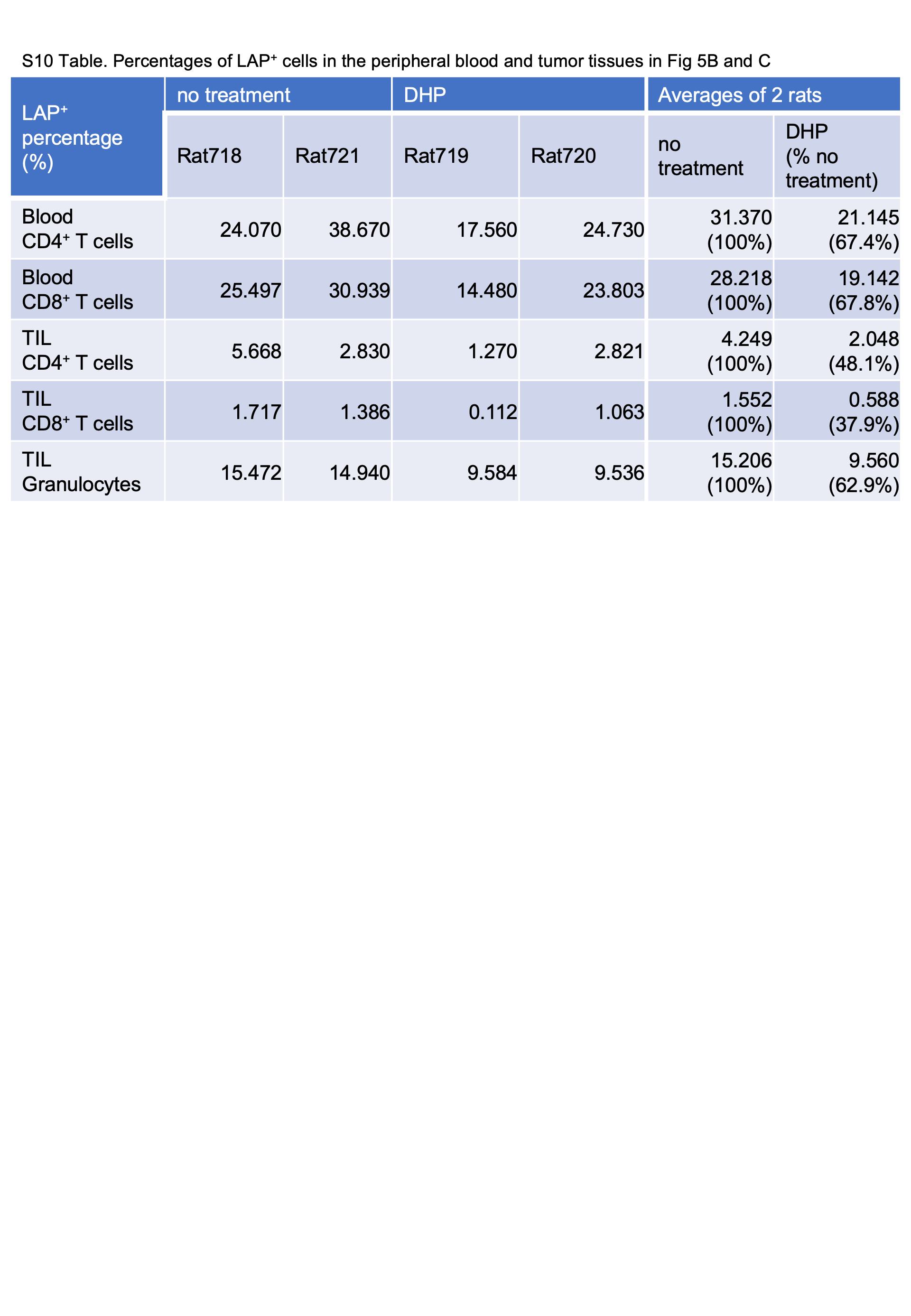

Supplement: S10 Table — (TIFF) [file pone.0305153.s010.tiff]

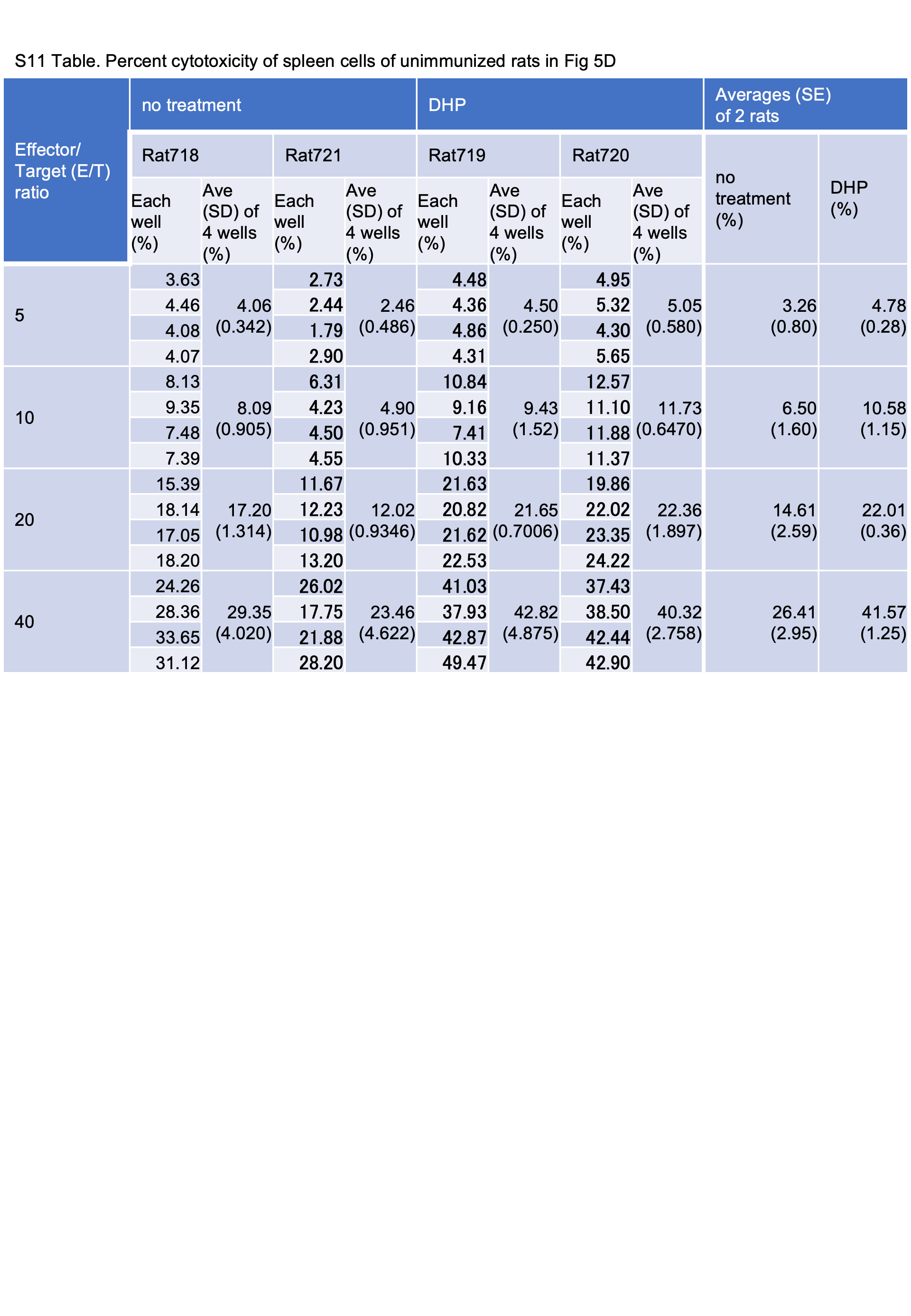

Supplement: S11 Table — (TIFF) [file pone.0305153.s011.tiff]

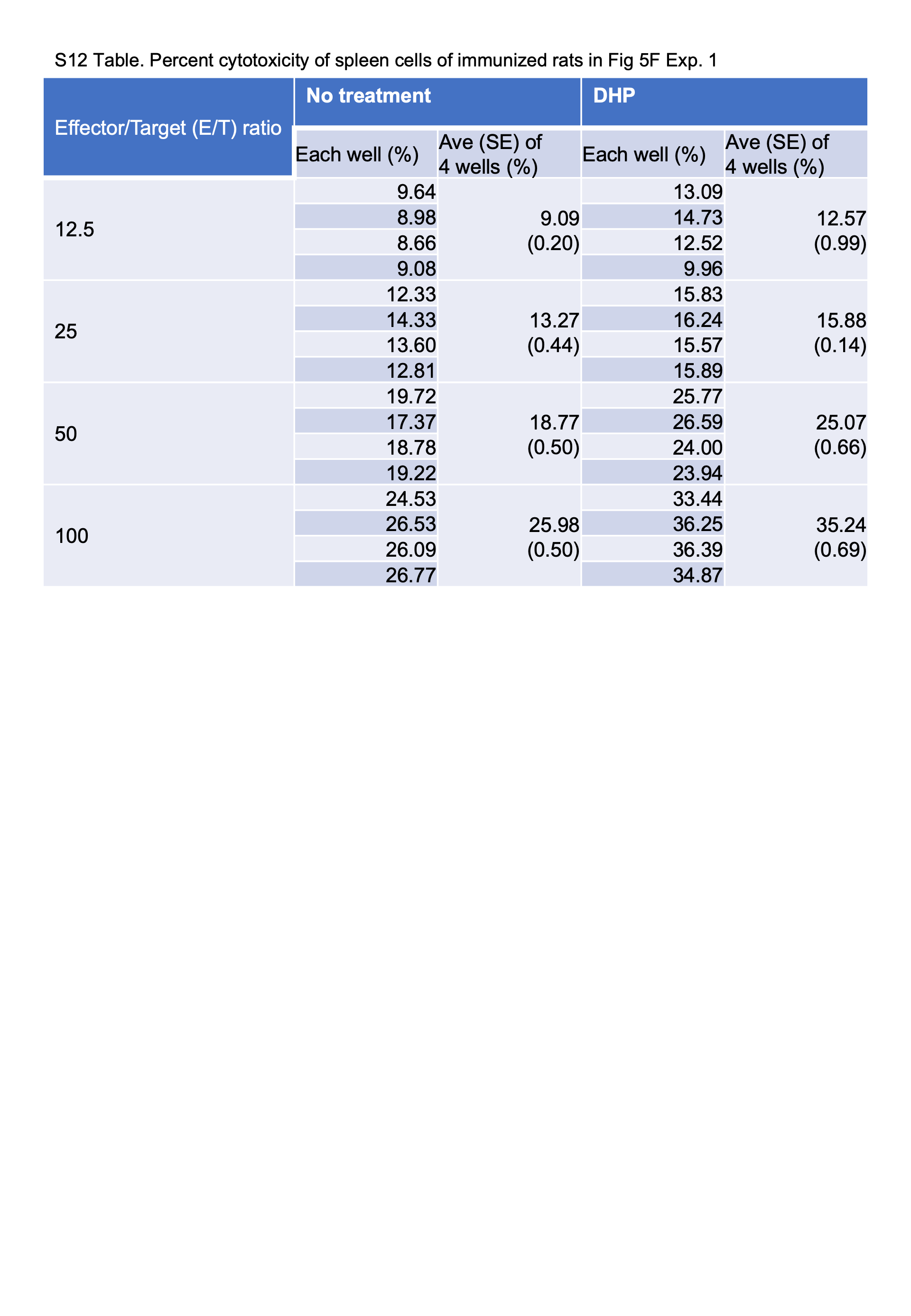

Supplement: S12 Table — (TIFF) [file pone.0305153.s012.tiff]

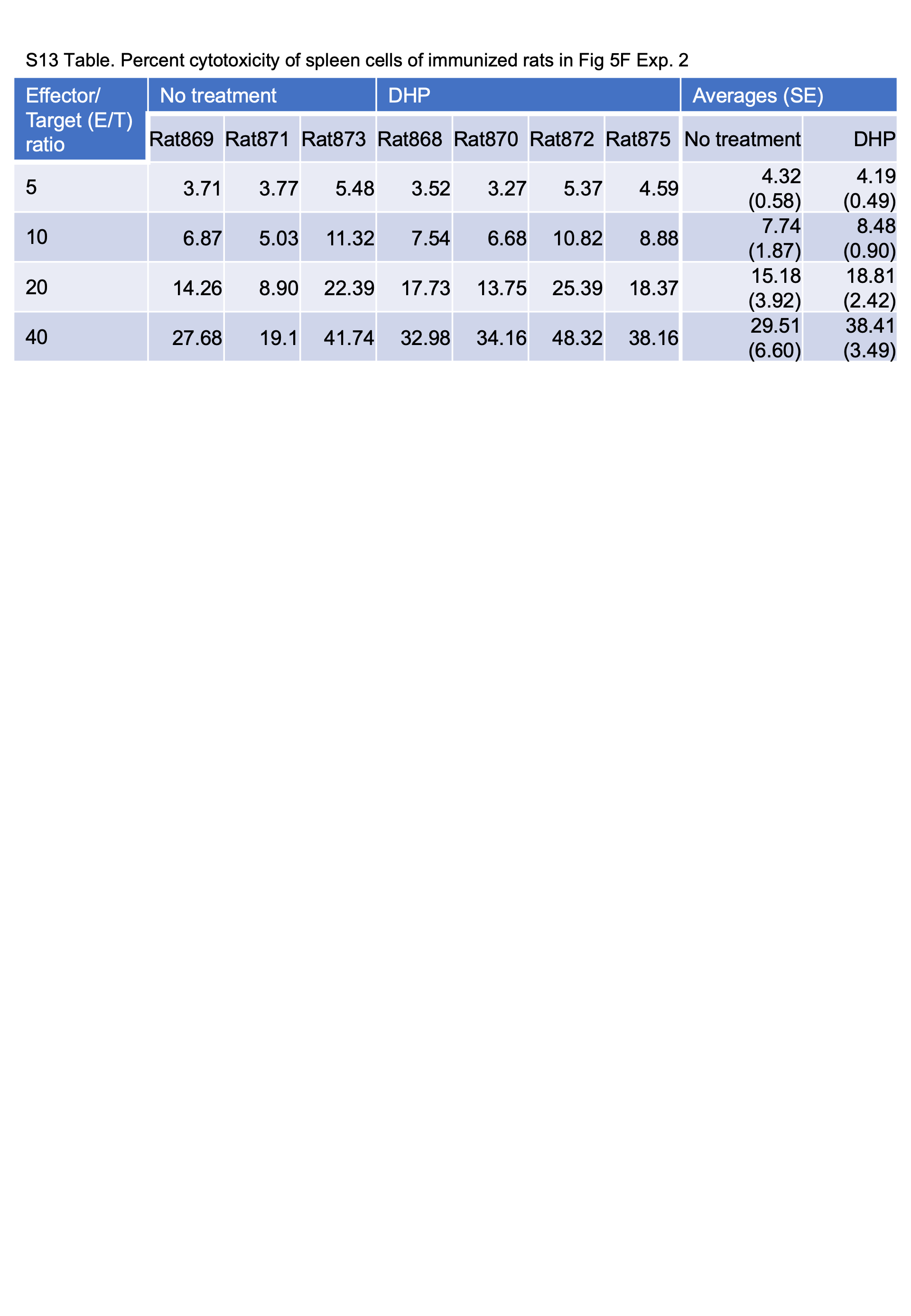

Supplement: S13 Table — (TIFF) [file pone.0305153.s013.tiff]

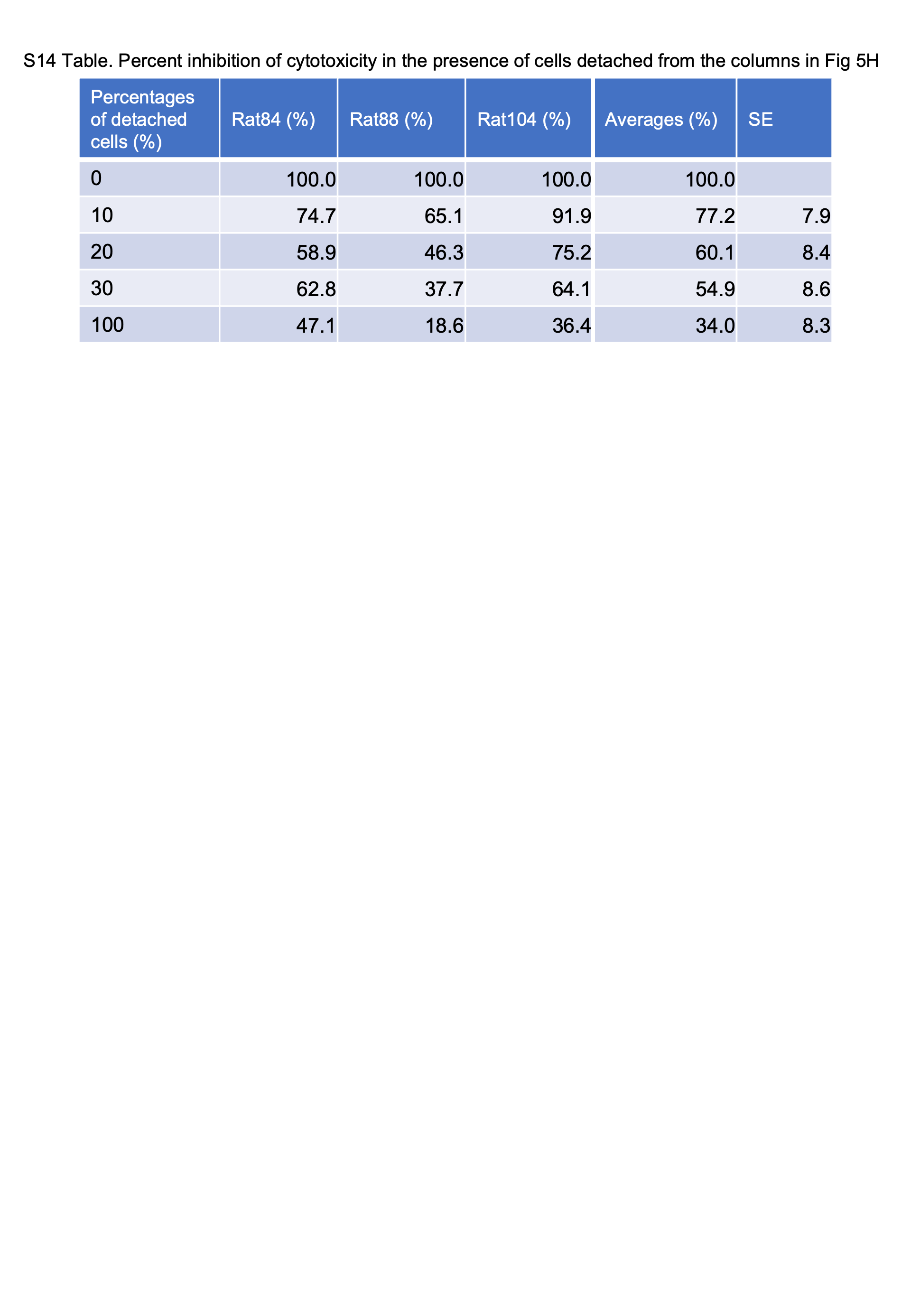

Supplement: S14 Table — (TIFF) [file pone.0305153.s014.tiff]

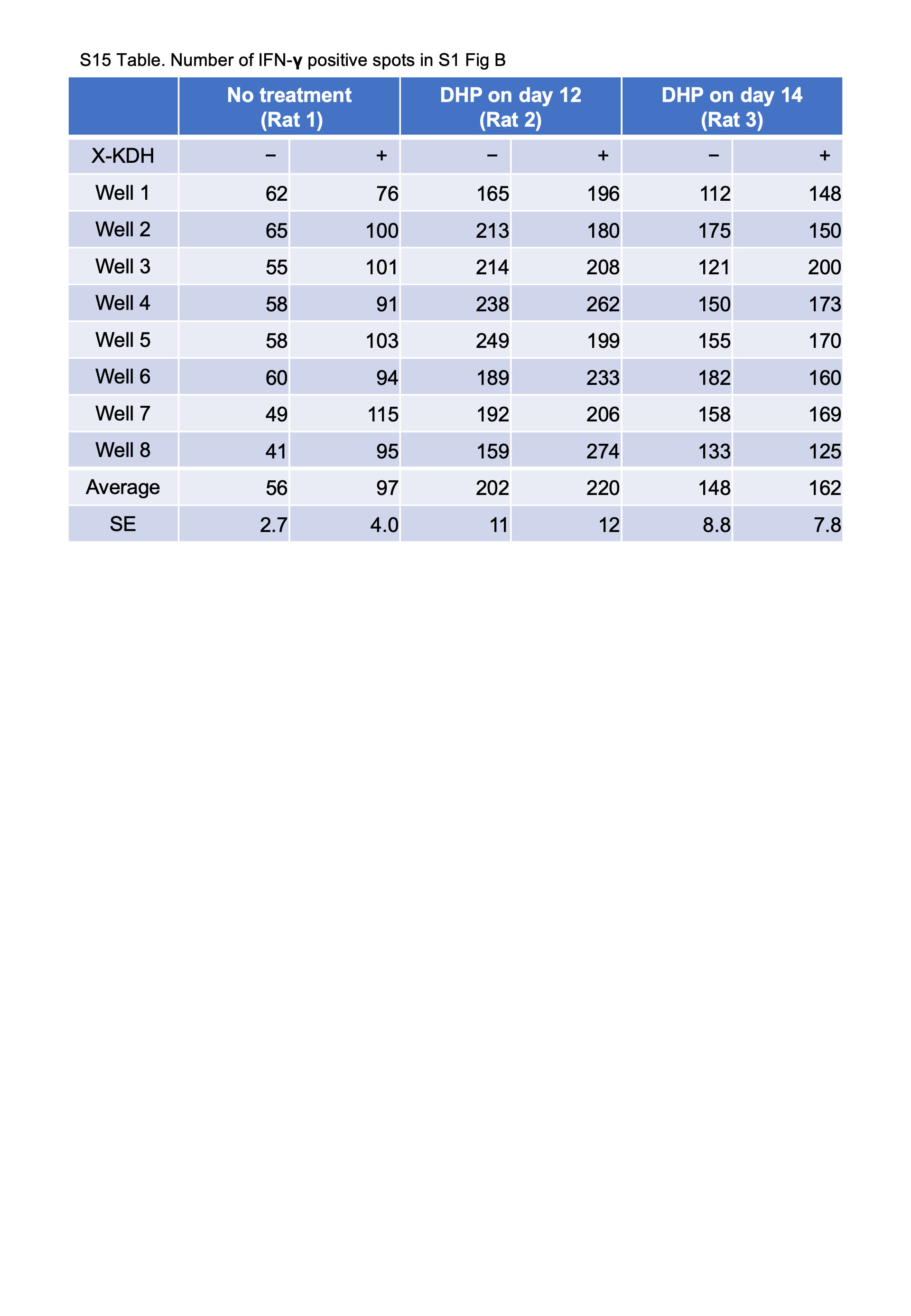

Supplement: S15 Table — (TIFF) [file pone.0305153.s015.tiff]

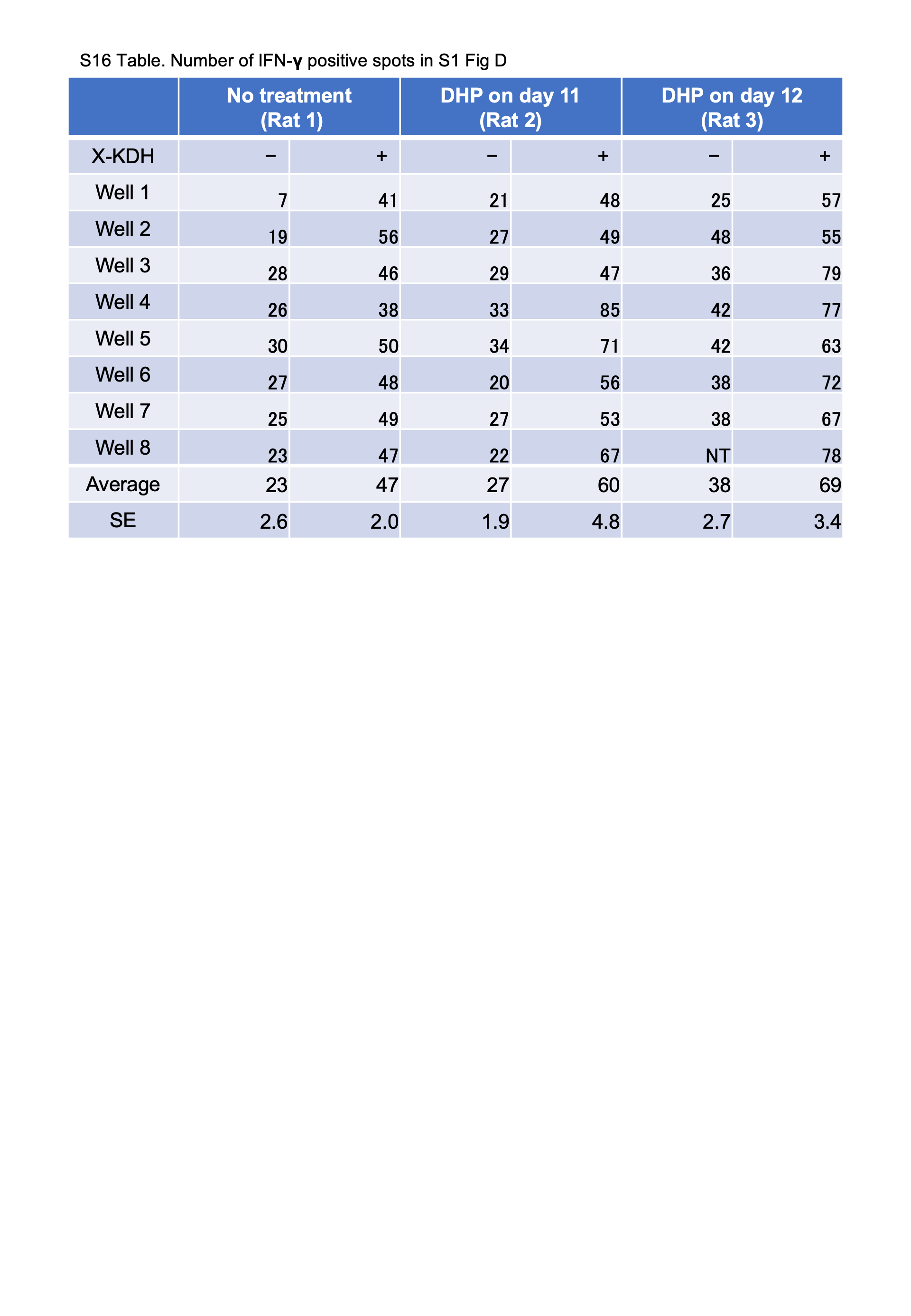

Supplement: S16 Table — (TIFF) [file pone.0305153.s016.tiff]

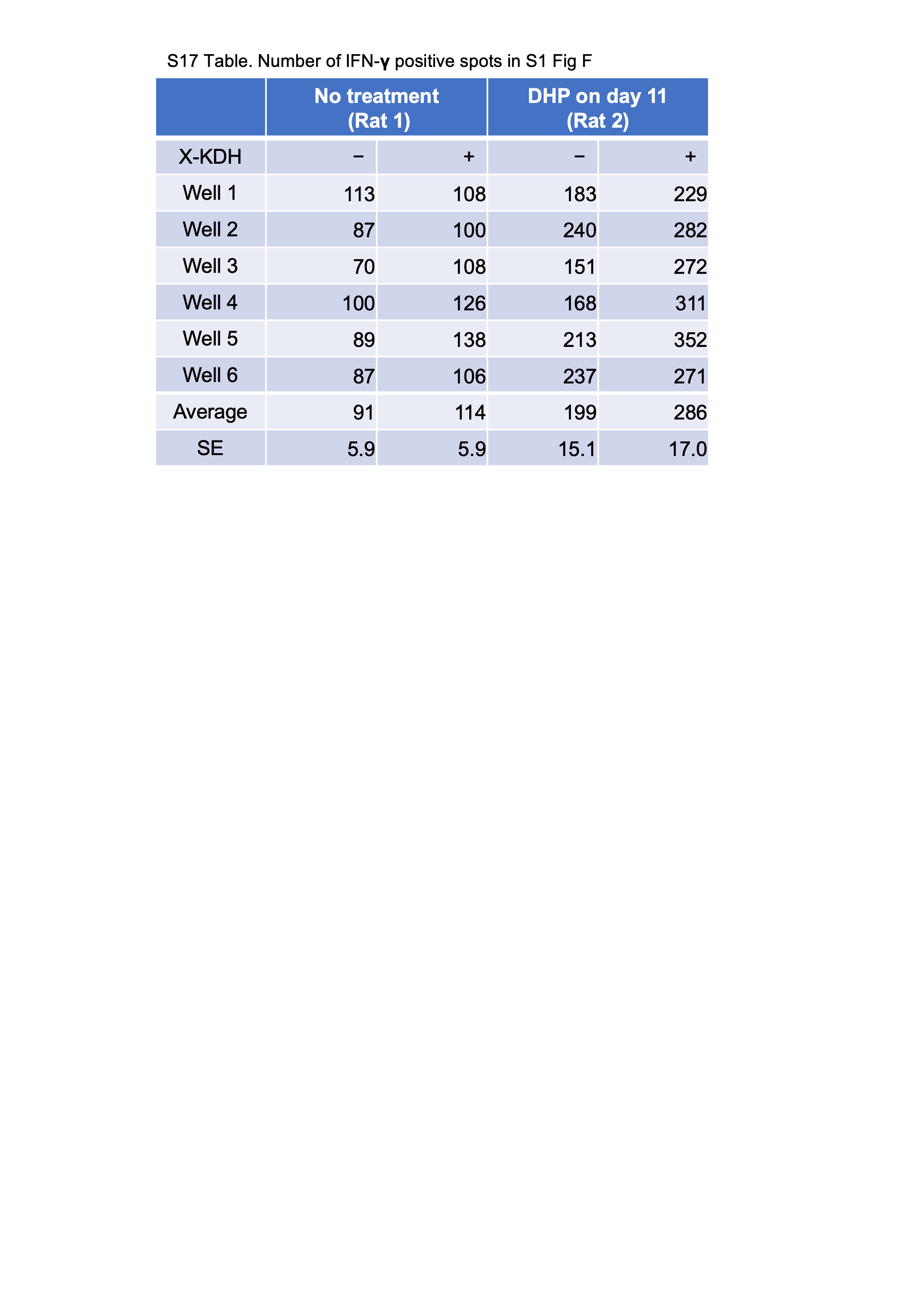

Supplement: S17 Table — (TIFF) [file pone.0305153.s017.tiff]

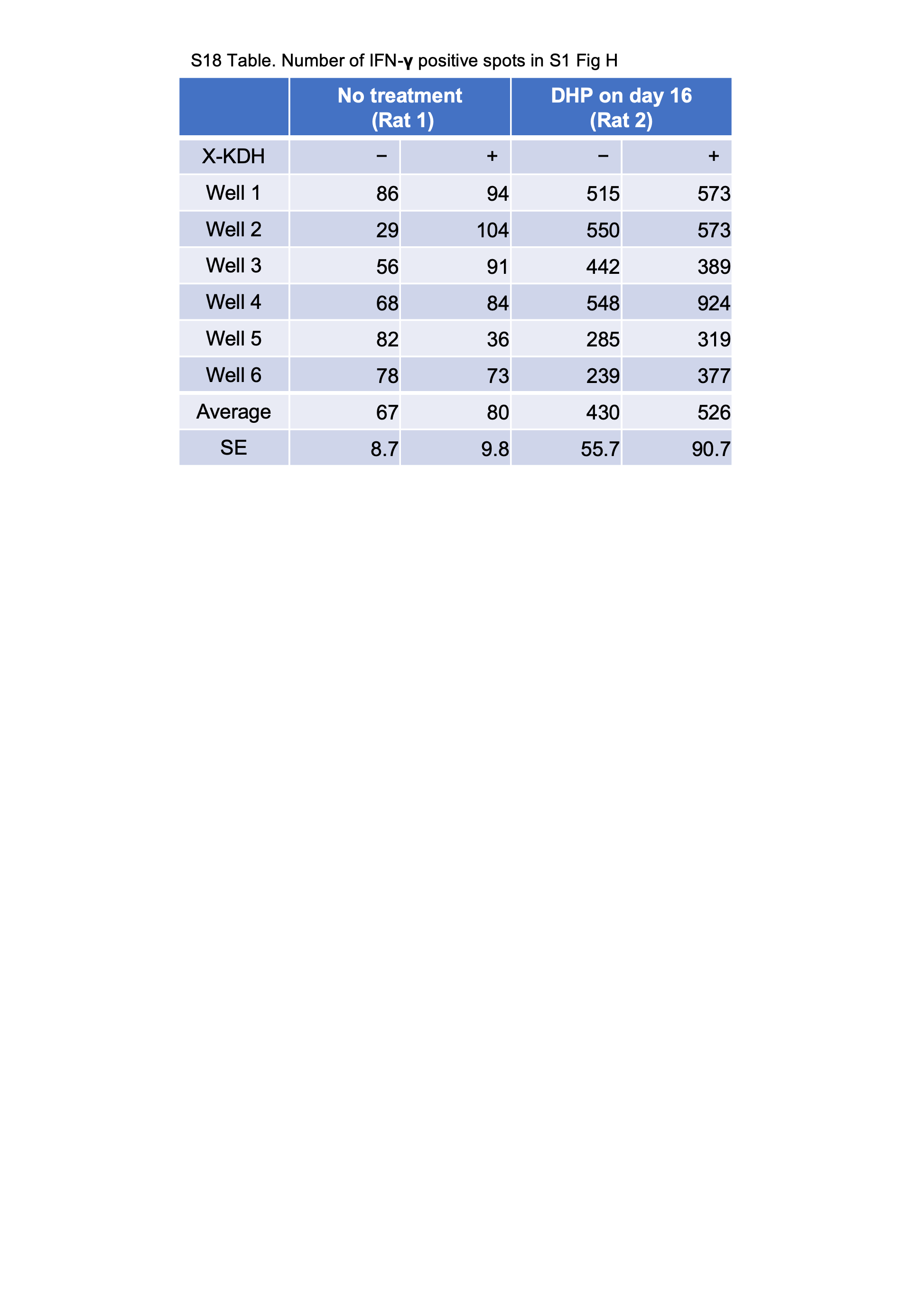

Supplement: S18 Table — (TIFF) [file pone.0305153.s018.tiff]

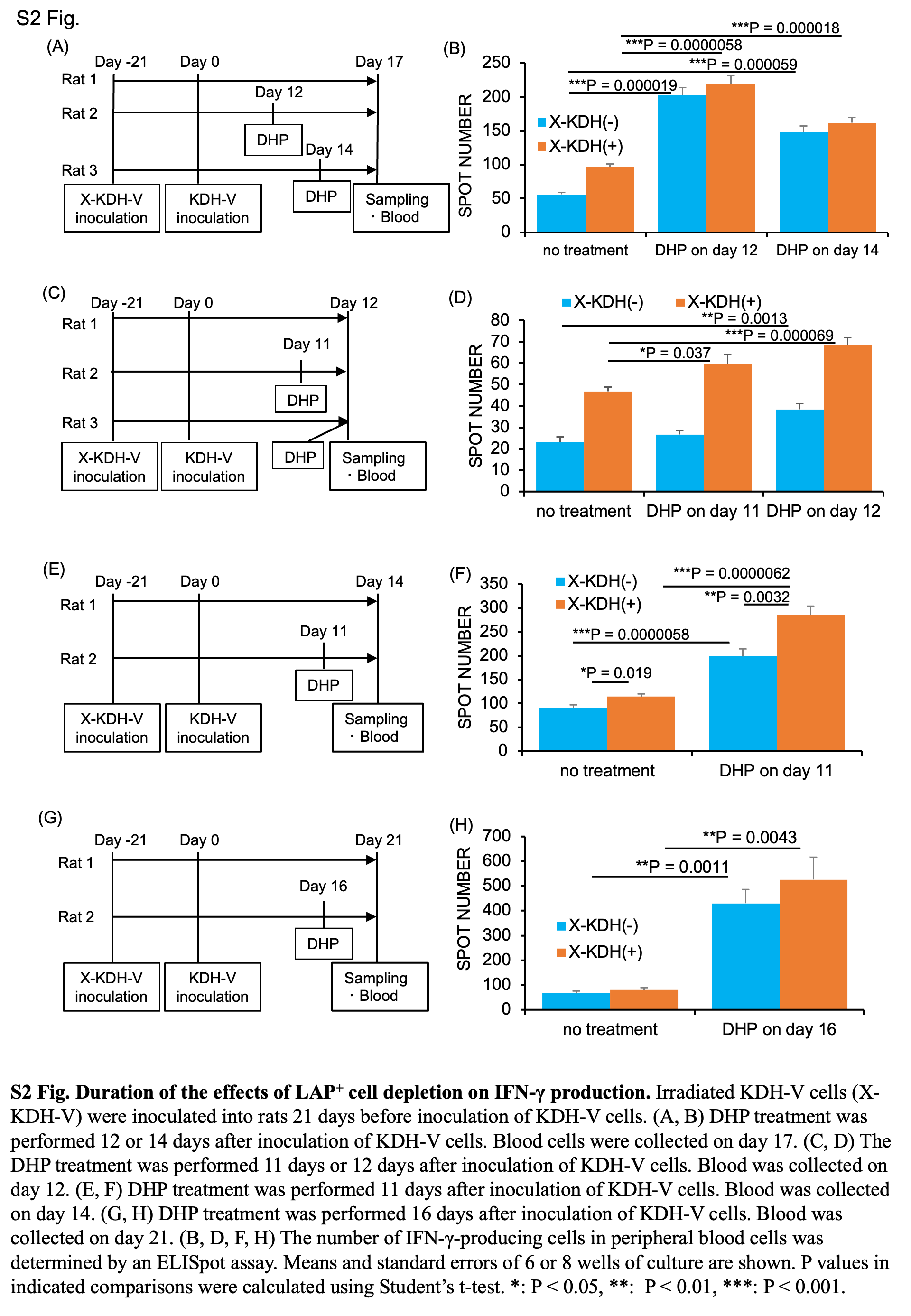

Supplement: S2 Fig — Irradiated KDH-V cells (X-KDH-V) were inoculated into rats 21 days before inoculation of KDH-V cells. (A, B) DHP treatment was performed 12 or 14 days after inoculation of KDH-V cells. Blood cells were collected on day 17. (C, D) The DHP treatment was performed 11 days or 12 days after inoculation of KDH-V cells. Blood was collected on day 12. (E, F) DHP treatment was performed 11 days after inoculation of KDH-V cells. Blood was collected on day 14. (G, H) DHP treatment was performed 16 days after inoculation of KDH-V cells. Blood was collected on day 21. (B, D, F, H) The number of IFN-γ-producing cells in peripheral blood cells was determined by an ELISpot assay. Means and standard errors of 6 or 8 wells of culture are shown. P values in indicated comparisons were calculated using Student’s t-test. * : P < 0.05, **: P < 0.01, ***: P < 0.001. (TIFF) [file pone.0305153.s020.tiff]

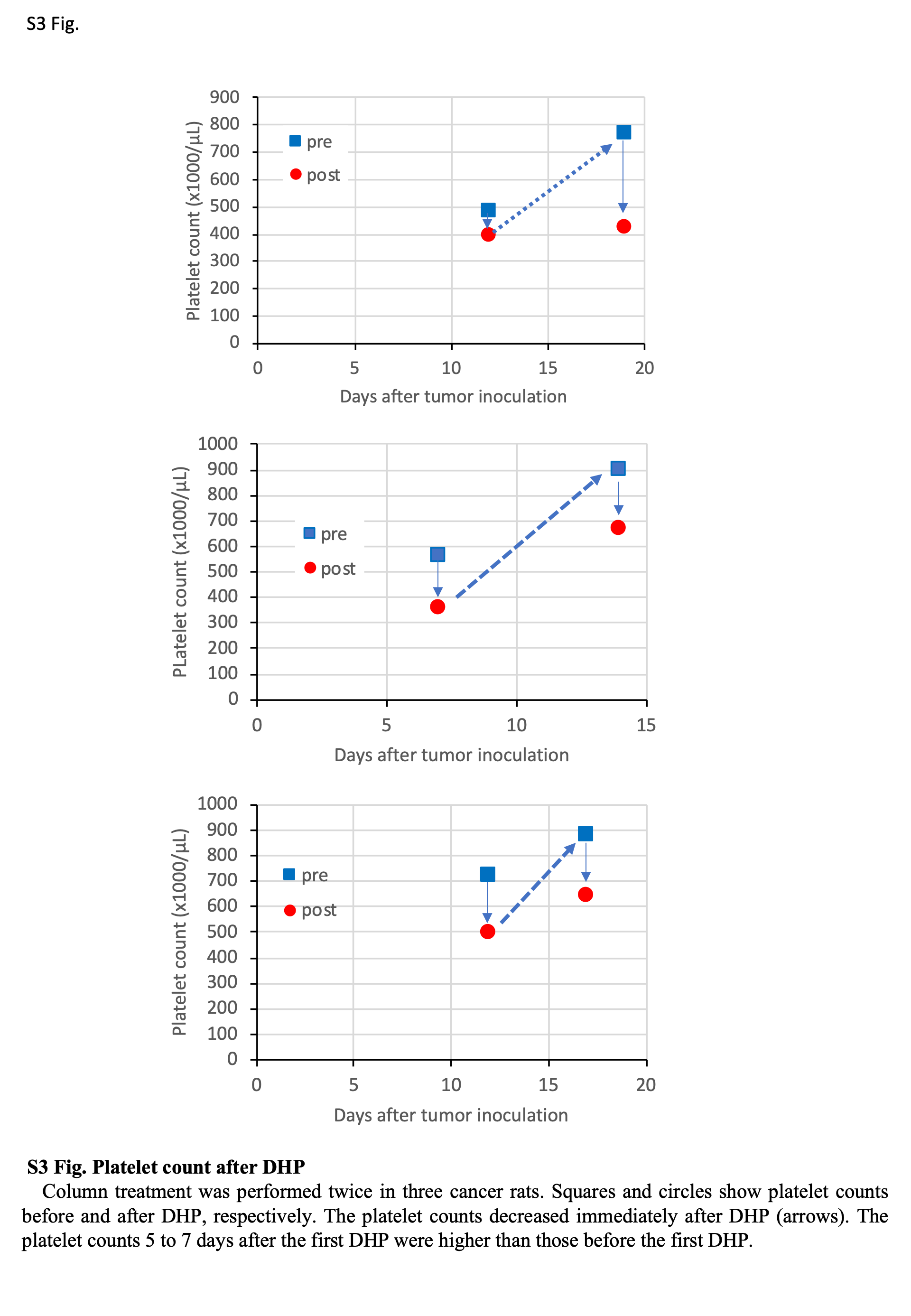

Supplement: S3 Fig — Column treatment was performed twice in three cancer rats. Squares and circles show platelet counts before and after DHP, respectively. The platelet counts decreased immediately after DHP (arrows). The platelet counts 5 to 7 days after the first DHP were higher than those before the first DHP. (TIFF) [file pone.0305153.s021.tiff]
