# Supplementary material for: A hemoperfusion column selectively adsorbs LAP+ lymphocytes to improve anti-tumor immunity and survival of tumor-bearing rats
Source: PLoS One. 2025 Mar 7;20(3):e0305153. doi: 10.1371/journal.pone.0305153 (PMC11888139; doi:10.1371/journal.pone.0305153)
Supplement: S1 Fig — (A) IR spectrum (KBr tablet); A Prestige-21 Fourier transform infrared spectrophotometer (Shimadzu Corporation, Kyoto, Japan) was used in the Central Research Laboratory in the Shiga University of Medical Science. Peaks of polysulfone skeleton were detected at 2968, 1579, 1487, and 1248 cm-1 (arrow heads). The carbonyl (C = 0) stretch of amidomethyl group was detected at 1671 cm-1 (arrow). (B) NMR spectrum (Chloroform-D); A JNM-ECZ400S NMR spectrometer (JEOL Ltd., Tokyo, Japan) was used in the Central Research Laboratory in the Shiga University of Medical Science. Levels of isopropylidene hydrogen of polysulfone skeleton (6H) (a), a peak of CH2 in chloroacetyl group (b), peaks of CH2 in an aminobenzyl group (c), and aromatic hydrogen (left peaks) were 1.66 ppm (singlet), 3.8 ppm (singlet), 4.35 ppm (doublet), and 6.8 – 7.9 ppm, respectively. (PDF) [file pone.0305153.s019.pdf]

S1 Fig

A

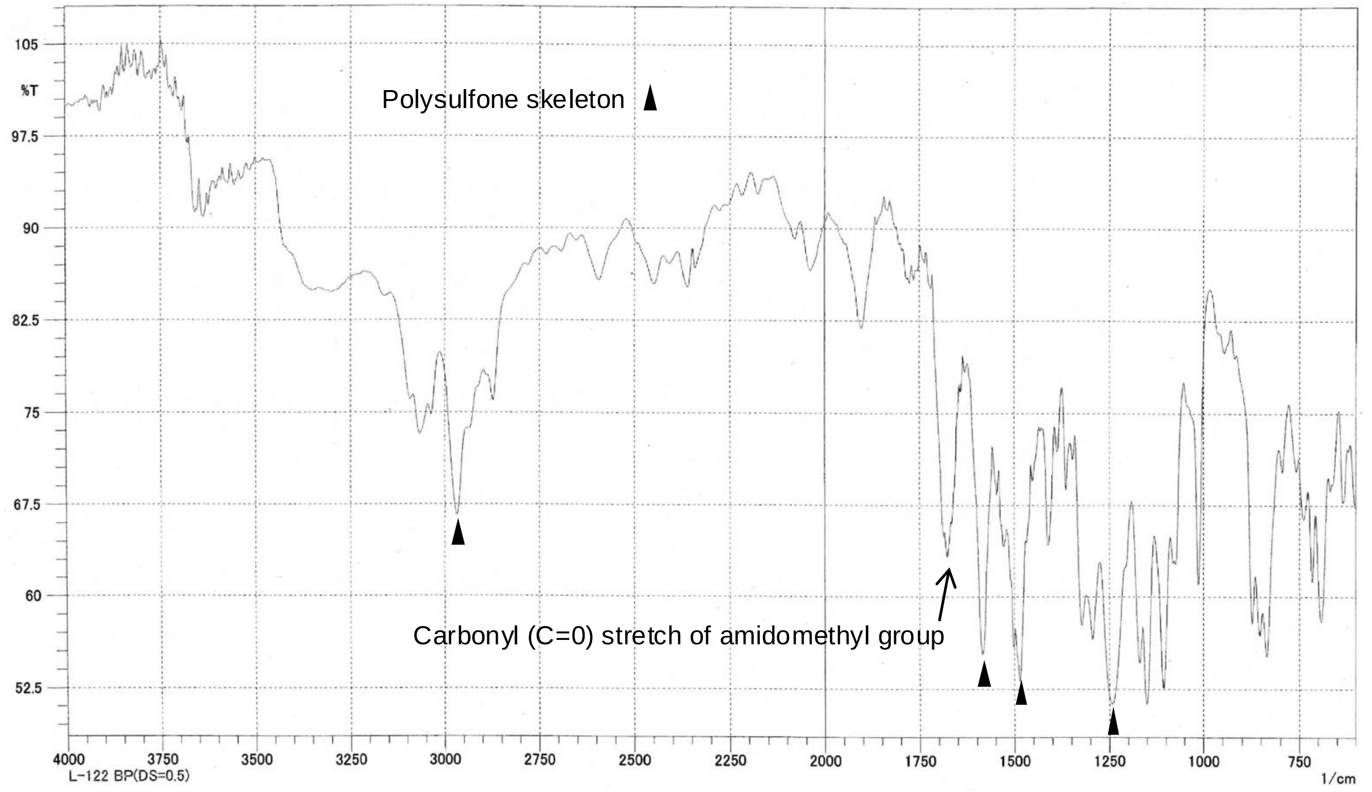

B

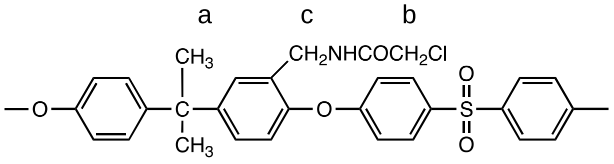

a: iso-propylidene-hydrogen 1.66 ppm
b: acetyl-hydrogen 3.80 ppm
c: benzyl-hydrogen 4.35 ppm

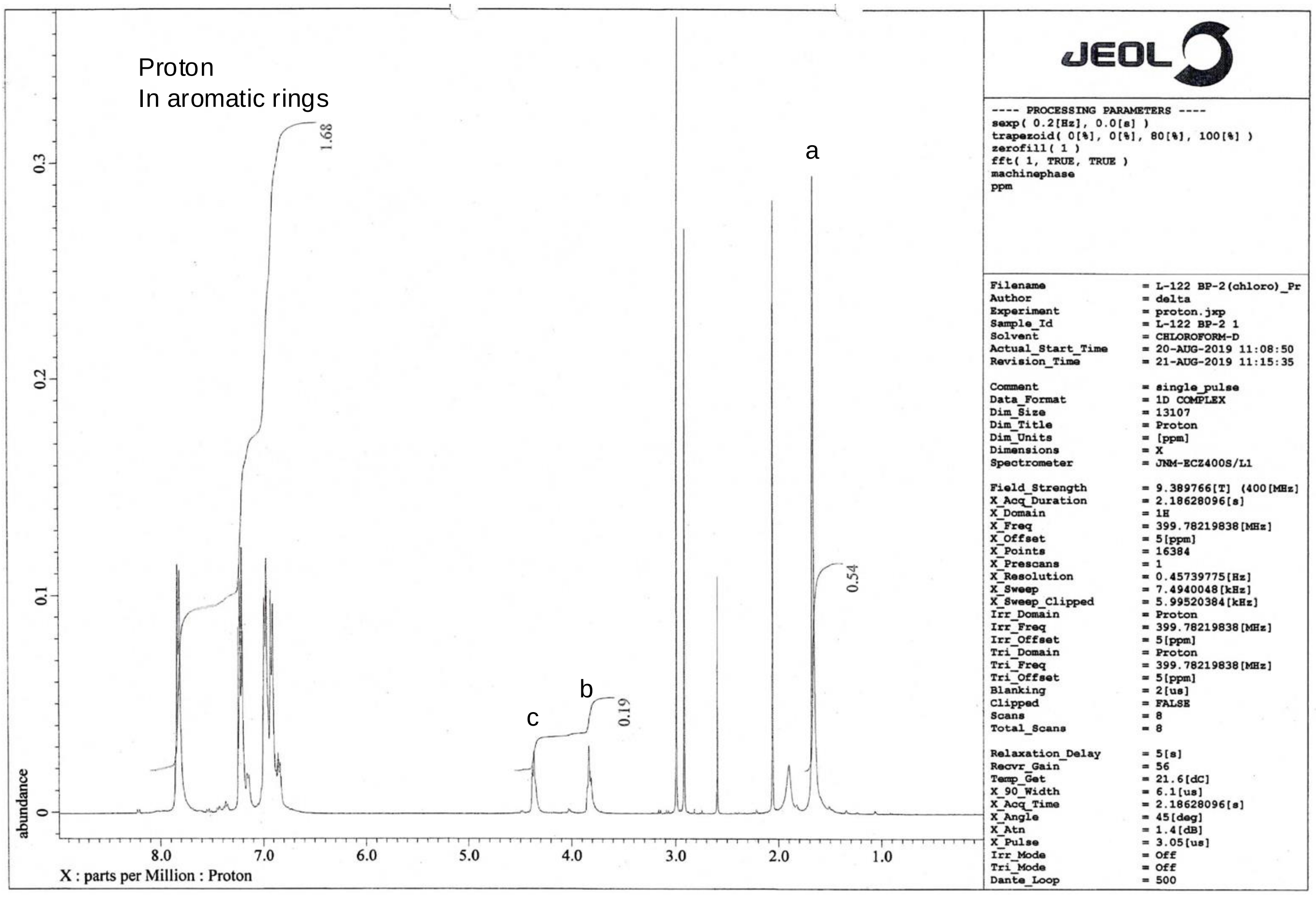

S1 Fig. IR and NMR spectrum analysis of chemical structure of absorbents

(A) IR spectrum (KBr tablet); A Prestige-21 Fourier transform infrared spectrophotometer (Shimadzu Corporation, Kyoto, Japan) was used in the Central Research Laboratory in the Shiga University of Medical Science. Peaks of polysulfone skeleton were detected at 2968, 1579, 1487, and 1248 cm^{-1} (arrow heads). The carbonyl (C=0) stretch of amidomethyl group was detected at 1671 cm^{-1} (arrow).
(B) NMR spectrum (Chloroform-D); A JNM-ECZ400S NMR spectrometer (JEOL Ltd., Tokyo, Japan) was used in the Central Research Laboratory in the Shiga University of Medical Science. Levels of isopropylidene hydrogen of polysulfone skeleton (6H) (a), a peak of CH_2 in chloroacetyl group (b), peaks of CH_2 in an aminobenzyl group (c), and aromatic hydrogen (left peaks) were 1.66 ppm (singlet), 3.8 ppm (singlet), 4.35 ppm (doublet), and 6.8 – 7.9 ppm, respectively.
